# Hereditary Thrombotic Thrombocytopenic Purpura Associated With Recurrent Strokes and Prominent Nervous System Involvement in a Young Chinese Female

**DOI:** 10.1002/ccr3.72102

**Published:** 2026-03-05

**Authors:** Wanying Liu, Jiaying Wu, Xi Ming, Qi Zhang, Delian Zhou, Rubing Zheng, Mi Zhou, Zhen Shang, Liting Chen, Xiaojian Zhu, Yi Xiao

**Affiliations:** ^1^ Department of Hematology, Tongji Hospital, Tongji Medical College Huazhong University of Science and Technology Wuhan Hubei China

**Keywords:** ADAMTS13, hereditary, stroke, thrombotic thrombocytopenic purpura

## Abstract

Hereditary thrombotic thrombocytopenic purpura (TTP) is a rare autosomal recessive inherited disease caused by an ADAMTS1 gene mutation, resulting in absence or severe deficiency of plasma ADAMTS13 activity. The common causes include infection, inflammation, or pregnancy. Here, we present a case involving a 30 year old female with hereditary TTP, identified as compound heterozygous mutations of ADAMTS13 c.1045C > T (p.Arg349Cys) and c.2411G > A (p.Cys804Tyr). Our findings suggest that patients with these mutations are prone to recurrent strokes and exhibit prominent neurological symptoms.

## Key Clinical Message

We present a case involving a 30‐year‐old female with hereditary TTP, identified as compound heterozygous mutations of ADAMTS13 c.1045C > T (p.Arg349Cys) and c.2411G > A (p.Cys804Tyr). Our case findings suggest that patients with these mutations are prone to recurrent strokes and exhibit prominent neurological symptoms.

## 1 Introduction

Hereditary thrombotic thrombocytopenic purpura (TTP) is a rare autosomal recessive inherited disease involving the ADMATS13 gene. Common causes of TTP include infection, inflammation or pregnancy. However, hereditary TTP occurs mostly in children and pregnant women, accounting for only 5% of all total cases of TTP [1].

Homozygous or double heterozygous mutations of theADAMTS13 gene, result in plasma ADAMTS13 activity absence or severe deficiency. ADAMTS13 is located on chromatin 9q34, contains 29 exons, and encodes a 1427 amino acid multidomain protein [2]. The most common types of mutations are nonsense, followed by missense, frameshift insertions, or deletions, which can be distributed throughout the gene [3].

Reduced or absent ADAMTS13 activity in the plasma results in the failure of timely degradation of abnormally released von Willebrand factor (VWF) multimers, which can spontaneously bind to platelets, leading to microvascular thrombosis and subsequent organ dysfunction [4, 5]. Most Hereditary TTP cases are diagnosed during childhood or early adolescence after the first acute episode. Here, we present a case of hereditary TTP in a 30 year old female, which resulted from a compound heterozygous mutation of ADAMTS13 c.1045C > T (p.Arg349Cys) and c.2411G > A(p.Cys 804Tyr).

## 2 Case History/Examination

A 30‐year‐old Chinese female was transferred to our hospital from a local emergency department where she presented for slurred speech and sudden numbness and weakness of the right upper and lower extremities while resting. Coughing while drinking water and dysphagia were also present. The patient was immediately taken to the local emergency department by her family. Cerebral magnetic resonance imaging (MRI) revealed acute infarction in the left cerebellar hemisphere and left temporo‐occipital lobe. Thrombolysis was not performed due to a low platelet count, and she was transferred to our hospital on September 21, 2023.

The patient had a previous stroke in 2011, which occurred at 18 years of age. This was characterized by right temporo‐occipital infarction with left upper and lower limb weakness and paralysis of the left facial and tongue muscles. Paroxysmal seizures occurred at a frequency of 3–4 times per year after the cerebral infarction, which were treated with maintenance levetiracetam and oxcarbazepine. She was diagnosed with immune thrombocytopenia (ITP) at a local hospital in 2012 and was prescribed a long‐term prednisone at 50 mg/day. At ages 19 and 29, she had spontaneous abortions due to fetal asphyxia at 16 weeks gestation. Her sister had a history of anemia. No other family members have similar clinical symptoms.

## 3 Methods (Differential Diagnosis, Investigations and Treatment)

On initial presentation, body temperature was 36.5°C, pulse was 89/min, respiratory rate was 20/min, blood pressure was 119/72 mmHg. She was able to cooperate during the physical examination, and consciousness was clear; however, her mood was depressed. Ecchymosis was observed on her left lower limb, but no other lesions were present here or on other body surfaces. Lung sounds were clear, without dry or moist rales. Superficial lymph nodes were not palpably swollen. Bilateral pupils were large and round, with a diameter of about 3 mm, and sensitive to light reflex; extraocular movement was intact bilaterally, without significant nystagmus. Bilateral frontal striae were symmetrical, but no tongue extension was present, and the left nasolabial groove was shallow. Muscle strength of the left upper limb was III+, and IV in the left lower limb; and IV for the right upper and lower limbs. The left pathological sign was positive, but Kernig's sign was negative. Laboratory results presented in Table 1, revealed microangiopathic hemolytic anemia and thrombocytopenia, with a hemoglobin concentration of 60.0 g/L and a platelet count of 32 × 10^9^/L. Renal dysfunction was also present. Urinalysis revealed occult blood (2+) and proteins (3+). The ADAMTS13 activity was below 5%, and no inhibitors were detected. The ADAMTS13 gene was sequenced. Direct sequencing of ADAMTS13 revealed two heterozygous mutations in the coding region, consisting of two missense mutations of p.Arg349Cys and p.Cys804Tyr. Bone marrow cytology revealed three lines of proliferative bone marrow with fragmented red blood cells accounting for 10% of the total.

**TABLE 1:** Laboratory parameters of the patient.

Laboratory examination	Value	Reference range
Hematology assays		
White blood cell (×10^9^/L)	**12.20**	3.5–9.5
Neutrophils (%)	**84.1**	40.0–75.0
Lymphocytes (%)	**9.6**	20.0–50.0
Hemoglobin (g/L)	**63.0**	115.0–150.0
Platelets (×10^9^/L)	**24.0**	125.0–350.0
Hypersensitive C‐reactive protein (mg/L)	**6.6**	< 1
Thrombosis and hemostasis assays		
PT (s)	14.0	11.5–14.5
Prothrombin activity (%)	85.0	80.0–135.0
INR	1.10	0.80–1.20
Fibrinogen (g/L)	3.67	2.00–4.00
APTT (s)	34.6	29.0–42.0
TT (s)	17.9	14.0–19.0
D‐dimer (μg/mL FEU)	0.44	< 0.5
Hepatic analysis		
Total bilirubin (μmol/L)	**28.2**	≤ 21
Direct bilirubin (μmol/L)	**8.8**	≤ 8.0
Indirect bilirubin (μmol/L)	**19.4**	≤ 12.9
Alanine aminotransferase (U/L)	7	≤ 33
Aspartate transaminase (U/L)	30	≤ 32
Lactic dehydrogenase (U/L)	**857**	135–241
Renal analysis		
Urea (mmol/L)	**10.40**	2.6–7.5
Creatinine (μmol/L)	**143**	45–84
Uric acid (μmol/L)	**809.0**	142.8–339.2
eGFR (mL/min/1.73 m^2^)	**42.4**	> 90
Urinalysis		
Urine protein	**3+**	Negative
Erythrocyte	**2+**	Negative
ADAMTS13 activity (%)	**< 5.0**	65.0–135.0
ADAMTS13 inhibitor	Negative	Negative
Other tests		
Haptoglobin (g/L)	**< 0.06**	0.36–1.95
Free hemoglobin (mg/L)	**125.90**	< 40.00
Direct anti‐human globulin test	Negative	Negative
Hypersensitive cardiac troponin I (pg/mL)	**372.5**	≤ 15.6
Hepatitis B virus	Negative	Negative
Hepatitis C virus	Negative	Negative
*Treponema pallidum*	Negative	Negative

*Note:* Bold Values indicate values outside the expected range.

Abbreviations: APTT, activated partial thromboplastin time; INR, international normalized ratio; PT, prothrombin time; TT, thrombin time.

## 4 Conclusion and Results (Outcome and Follow‐Up)

A diagnosis of hereditary thrombotic thrombocytopenic purpura was definitively made based on the above clinical and laboratory findings. We administer fresh frozen plasma daily to the patient at a dose of 10–15 mL/kg, and perform plasma exchange every 2 days according to a standard of 30–40 mL/kg. The typical duration for daily plasma infusion is 30 min, while plasma exchange usually lasts about 4 h. ADAMTS13 activity increased to 40.2%, and platelet count returned to normal (134 × 10^9^/L) after plasma treatment. Vital signs remained stable and the patient was discharged on hospital day 7. Prednisolone 8 mg/day was continued outside the hospital, and monthly fresh frozen plasma infusion (800–1000 mL) therapy was recommended. Three months after discharge, the patient was readmitted due to hearing loss. Routine blood tests showed a decrease in the platelet count (34 × 10^9^/L), while blood biochemistry indicates lactate dehydrogenase at 774 U/L, and the ADAMTS13 level dropped to 3.3%. The patient had markedly elevated levels of thrombomodulin, a marker indicating vascular endothelial injury. To swiftly clear vWF from the body and alleviate further vascular damage, and to promptly supplement ADAMTS13, we initiated plasma exchange and plasma transfusion therapy. After therapy, the platelet count increased to 96 × 10^9^/L, and lactate dehydrogenase decreased to 283 U/L. The patient's symptoms and related indicators improved, and the patient was discharged 5 days later. During the most recent follow‐up call, we learned that the patient is in good general condition with no significant discomfort.

## 5 Discussion

The main manifestations of TTP include microvascular hemolytic anemia, thrombocytopenia, neuropsychiatric symptoms, fever, and kidney damage. TTP is a thrombotic microangiopathy related to a severe deficiency of ADAMTS13, leading to spontaneous formation of platelet thrombi responsible for mechanical hemolytic anemia, thrombocytopenia, and multi‐organ ischemia, often with brain involvement and corresponding neurological clinical manifestations (stroke, seizure, or confusion). Symptoms usually manifest in childhood or in young adult females during pregnancy.

Clinical presentations of hereditary TTP vary widely, and during childhood, it can often be misdiagnosed as primary ITP. Symptoms develop shortly after birth in some patients, whereas others remain asymptomatic until the second or third decade of life. Therefore, it is imperative to consider TTP (hereditary or acquired) in the differential diagnosis of cryptogenic stroke in young patients with thrombocytopenia. In our case, the patient exhibited the typical cerebrovascular and systemic complications and chronic renal dysfunction. The increased free hemoglobin, in combination with decreased haptoglobin, indicated hemolysis, while there was no evidence of autoimmune hemolytic anemia with a negative Coomb's assay. Disseminated intravascular coagulation was excluded based on normal APTT, PT, and fibrinogen levels. Finally, the definitive diagnosis of hereditary TTP relies on ADAMTS13 activity below 5%, absence of inhibitors, and identification of two heterozygous mutations in ADAMTS13 gene sequencing.

ADAMTS13 p.Arg349Cys heterozygous missense mutation results in an arginine to cysteine substitution on the 349th amino acid of the protein chain. This mutation has been reported in the gnomAD, ExAC, 1000G databases, but not the HGMD database. An ADAMTS13 p.Cys804Tyr heterozygous missense mutation was also detected and is associated with a cysteine to tyrosine substitution at the 804th position of the protein. This mutation has not been reported in the gnomAD, ExAC, 1000G databases, but has been reported in the HGMD database.

The International Thrombotic Thrombocytopenic Purpura Registry recruited 123 confirmed cases between 2006 and 2017. Stroke occurred in 21% of patients and transient ischemic attacks in 10%. Other neurological disorders included epileptic seizures (six patients), headache (five patients), and psychiatric conditions (depression, behavioral, and mental disorders; 20 patients). The most frequent mutation observed was ADAMTS13 c.4143_4144dupA (present on 60 of 246 alleles), followed by c.3178C>T (13 of 246 alleles) and c.577C>T (11 of 246 alleles) [6]. Based on interviews with 27 patients enrolled in the International Hereditary Thrombotic Thrombocytopenic Purpura Registry from 17 US states between April 2020 and July 2021, stroke was reported in 17 patients (63%) with a median age of 26 years (range, newborn to 58 years) for the first stroke. Three of the 12 patients were diagnosed at the time of their first stroke; the other nine patients were diagnosed with hereditary TTP 2 months to 19 years after their first stroke. Eleven of the 17 patients had residual symptoms, and seven had recurrent strokes [7]. Similarly, a meta‐analysis including 217 patients with hereditary TTP showed that 62 (29%) had a stroke. Thirteen (21%) of the 62 patients with stroke were 10 years old or younger [8]. Additionally, of 15 pregnancies with TTP, eight cases resulted in stillbirth or death shortly after birth. It is believed that the lack of ADAMTS13 enzyme activity in maternal plasma is harmful to both the mother and fetus [9]. The variant identified in the ADAMTS13 gene in our case may have had a specific functional impact on the placenta, leading to recurrent fetal death during pregnancy.

To date, fresh frozen plasma infusion remains the most commonly used method for preventing and treating symptoms related to hereditary TTP caused by ADAMTS13 mutations [10]. Plasma exchange may also be used in hereditary TTP. Patients with suspected TTP are often admitted with acute and severe symptoms, but genetic sequencing results are not immediately available. Therefore, initial diagnosis of acquired TTP or hereditary TTP relies largely on ADAMTS13 activity, which is not always definitive. Thus, the standard initial treatment during the acute phase for patients without a confirmed diagnosis of TTP remains plasma exchange. Functional ADAMTS13 can be supplemented and high molecular weight vWF can be removed through plasma exchange. The dosage is typically 30–40 mL/kg per session. Both therapies lead to remarkable treatment effects in the acute phase by supplementing plasma enzyme activity and removing adverse factors that lead to vascular endothelial cell injury and platelet aggregation. Reduced ADAMTS13 activity at presentation of ischemic stroke is associated with worse neurological deficits and increased mortality [11]. Timely recognition of TTP and appropriate treatment will reduce the incidence of neurological sequelae and mortality in these patients. In non‐randomized studies, routine preventive treatment and prophylactic ADAMTS13 infusions significantly reduce the incidence of cerebrovascular events [10].

In summary, we report the case of a young female with hereditary TTP, which was identified as a compound heterozygous mutation of ADAMTS13, c.1045C > T and c.2411G > A. Differences in the phenotype and severity of congenital TTP may be the result of multiple environmental and genetic factors acting in concert. Our findings suggest that patients with these mutations are prone to recurrent stroke and exhibit prominent neurological symptoms.

## Author Contributions

**Wanying Liu:** data curation, writing – original draft. **Jiaying Wu:** data curation, writing – original draft. **Xi Ming:** data curation. **Qi Zhang:** data curation. **Delian Zhou:** data curation. **Rubing Zheng:** data curation. **Mi Zhou:** data curation. **Zhen Shang:** data curation. **Liting Chen:** data curation. **Xiaojian Zhu:** writing – review and editing. **Yi Xiao:** writing – review and editing.

## Funding

This work was supported by the National Natural Science Foundation of China (No. 82270183 and 82370196).

## Consent

Written informed consent was obtained from the patient to publish this report in accordance with the journal's patient consent policy.

## Conflicts of Interest

The authors declare no conflicts of interest.

## Data Availability

The data supporting the findings of this study are available from the corresponding author upon reasonable request.
